# Determinantes sociales y mortalidad por COVID-19: revisión sistemática del papel de la atención primaria

**DOI:** 10.1016/j.aprim.2026.103617

**Published:** 2026-09-23

**Authors:** Carlos Eduardo de Sousa Praxedes, Gonçalo Alves de Sousa Santinha, Sharmênia de Araújo Soares Nuto, Roberto Wagner Junior Freire, Kelen Gomes Ribeiro, Luiz Odorico Monteiro de Andrade

**Affiliations:** a Fundación Oswaldo Cruz, Eusébio, Ceará, Brasil; b GOVCOPP – Unidade de Investigação em Governança, Competitividade e Políticas Públicas, Universidade de Aveiro, Aveiro, Portugal; c Universidad Federal de Ceará, Fortaleza, Ceará, Brasil

**Keywords:** Determinantes sociales de la salud, Mortalidad, COVID-19, Atención primaria de salud, Equidad en salud, Social determinants of health, Mortality, COVID-19, Primary health care, Health equity

## Abstract

**Objetivos:**

Sintetizar la asociación entre determinantes sociales y mortalidad por COVID-19 y examinar el papel de la atención primaria.

**Diseño:**

Revisión sistemática registrada en PROSPERO (CRD42024502678) y reportada conforme a PRISMA 2020.

**Fuentes de datos:**

PubMed/MEDLINE, EMBASE, Web of Science y Scopus, en inglés, portugués o español.

**Selección de estudios:**

Estudios observacionales sobre determinantes sociales, mortalidad por COVID-19 y atención primaria.

**Extracción de datos:**

País, diseño, determinantes, papel de la atención primaria, desenlace y medidas de asociación.

**Resultados:**

Se incluyeron 24 de 259 registros; 14 fueron de Estados Unidos y 13 ecológicos. En una cohorte española, la renta < 18.000 € se asoció con mayor mortalidad (aOR 3,29; IC 95% 2,16-5,03), de acuerdo con otros estudios de privación. En 3.141 condados estadounidenses, el quintil de mayor vulnerabilidad social presentó mayor mortalidad (RR 2,42; IC 95% 2,22-2,64), con dirección concordante en otros análisis territoriales. En Georgia, el déficit de profesionales de atención primaria se asoció con mayor mortalidad (RR 1,74; IC 95% 1,00-3,00); en Texas, mayor densidad médica de atención primaria se asoció con menor probabilidad de registrar muertes (OR 0,93; IC 95% 0,89-0,99). Los efectos por raza/etnicidad variaron según el contexto. La heterogeneidad metodológica, el predominio ecológico y la concentración estadounidense limitan la generalización.

**Conclusiones:**

La mortalidad por COVID-19 estuvo socialmente estratificada y territorialmente modulada. La atención primaria mostró asociaciones compatibles con un efecto mitigador parcial, sin sustituir políticas redistributivas e intersectoriales.

## Abstract (en)

**Objectives:**

To synthesize associations between social determinants and COVID-19 mortality and examine the role of primary care.

**Design:**

Systematic review registered in PROSPERO (CRD42024502678) and reported according to PRISMA 2020.

**Data sources:**

PubMed/MEDLINE, EMBASE, Web of Science and Scopus, in English, Portuguese, or Spanish.

**Study selection:**

Observational studies addressing social determinants, COVID-19 mortality, and primary care.

**Data extraction:**

Country, design, determinants, role of primary care, outcome, and association measures.

**Results:**

Of 259 records, 24 studies were included; 14 were conducted in the US and 13 had an ecological design. In a Spanish cohort, income < € 18,000 was associated with higher mortality (aOR 3.29; 95% CI 2.16-5.03), consistent with other studies of deprivation. Across 3141 US counties, the highest social-vulnerability quintile had higher mortality (RR 2.42; 95% CI 2.22-2.64), with a similar direction in other territorial analyses. In Georgia, primary care professional shortage was associated with higher mortality (RR 1.74; 95% CI 1.00-3.00), whereas in Texas greater primary care physician density was associated with lower odds of recording a death (OR 0.93; 95% CI 0.89-0.99). Race/ethnicity-related effects varied by context. Methodological heterogeneity, predominance of ecological designs, and concentration of US studies limit generalizability.

**Conclusions:**

COVID-19 mortality was socially stratified and territorially modulated. Primary care showed associations compatible with a partial mitigating effect but does not replace redistributive and intersectoral policies.

## Introducción

La pandemia de COVID-19 mostró que los eventos epidémicos no son socialmente neutros. Desde la perspectiva de los determinantes sociales de la salud, las desigualdades de renta, vivienda, trabajo, protección social y racialización configuran exposiciones diferenciales, vulnerabilidad acumulada y capacidad desigual de respuesta frente a crisis sanitarias^1^, ^2^. Durante la pandemia, ese patrón se expresó en mayor mortalidad en territorios con privación material, segregación residencial, barreras geográficas y menor capacidad institucional^3^, ^4^, ^5^, ^6^, ^7^, ^8^, ^9^.

La producción científica sobre COVID-19 y desigualdad creció rápidamente, pero persiste heterogeneidad en el nivel de análisis, los indicadores sociales y la forma de incorporar la atención primaria de salud (APS). Algunos estudios la examinan mediante densidad profesional, infraestructura comunitaria, cobertura o acceso longitudinal; otros la tratan como contexto asistencial^5^, ^8^, ^10^, ^11^, ^12^, ^13^, ^14^. El objetivo de esta revisión fue sintetizar la evidencia sobre determinantes sociales y mortalidad por COVID-19, examinar el papel de la APS y valorar la certeza de la evidencia mediante GRADE^15^, ^16^.

## Material y métodos

Se realizó una revisión sistemática basada en un protocolo registrado en PROSPERO (CRD42024502678) y reportada conforme a PRISMA 2020^17^.

Estrategia de búsqueda y criterios de elegibilidad: se realizaron búsquedas en PubMed/MEDLINE, EMBASE, Web of Science y Scopus mediante descriptores controlados y términos libres relacionados con social determinants of health, mortality, COVID-19 y primary health care. Se incluyeron estudios observacionales (ecológicos, transversales, de cohorte y de casos y controles) que evaluaran determinantes sociales y mortalidad por COVID-19 e incluyeran o permitieran discutir el papel de la APS o del acceso a servicios. Se consideraron publicaciones en inglés, portugués y español, sin restricción de período. Se excluyeron editoriales, revisiones, relatos de caso y estudios fuera del protocolo.

La selección de estudios se realizó en 2 etapas: cribado por títulos y resúmenes y evaluación a texto completo, con apoyo de Rayyan^18^. Se extrajeron país, diseño, unidad de análisis, desenlace, determinantes, presencia directa o contextual de APS/acceso, medidas de asociación, IC 95% y valores de p cuando estuvieron disponibles.

### Síntesis y evaluación de la evidencia

La heterogeneidad de diseños, unidades de análisis, definiciones de exposición y medidas de mortalidad hizo inapropiado combinar cuantitativamente los resultados. Se realizó una síntesis narrativa estructurada por ejes analíticos y una presentación tabular de las medidas reportadas, sin metaanálisis. La certeza de la evidencia se valoró mediante GRADE aplicado a las proposiciones centrales de la revisión^15^, ^16^.

## Resultados

Se identificaron 259 registros. Tras eliminar 68 duplicados, se cribaron 191 títulos y resúmenes; 144 fueron excluidos. Se evaluaron 47 informes a texto completo y se excluyeron 23 por ausencia de mortalidad (n = 5), exposición ajena a los determinantes sociales (n = 8), ausencia de APS/acceso a servicios (n = 5) o tipo de estudio no elegible (n = 5). Finalmente, 24 estudios fueron incluidos (fig. 1).

**Figura 1:**
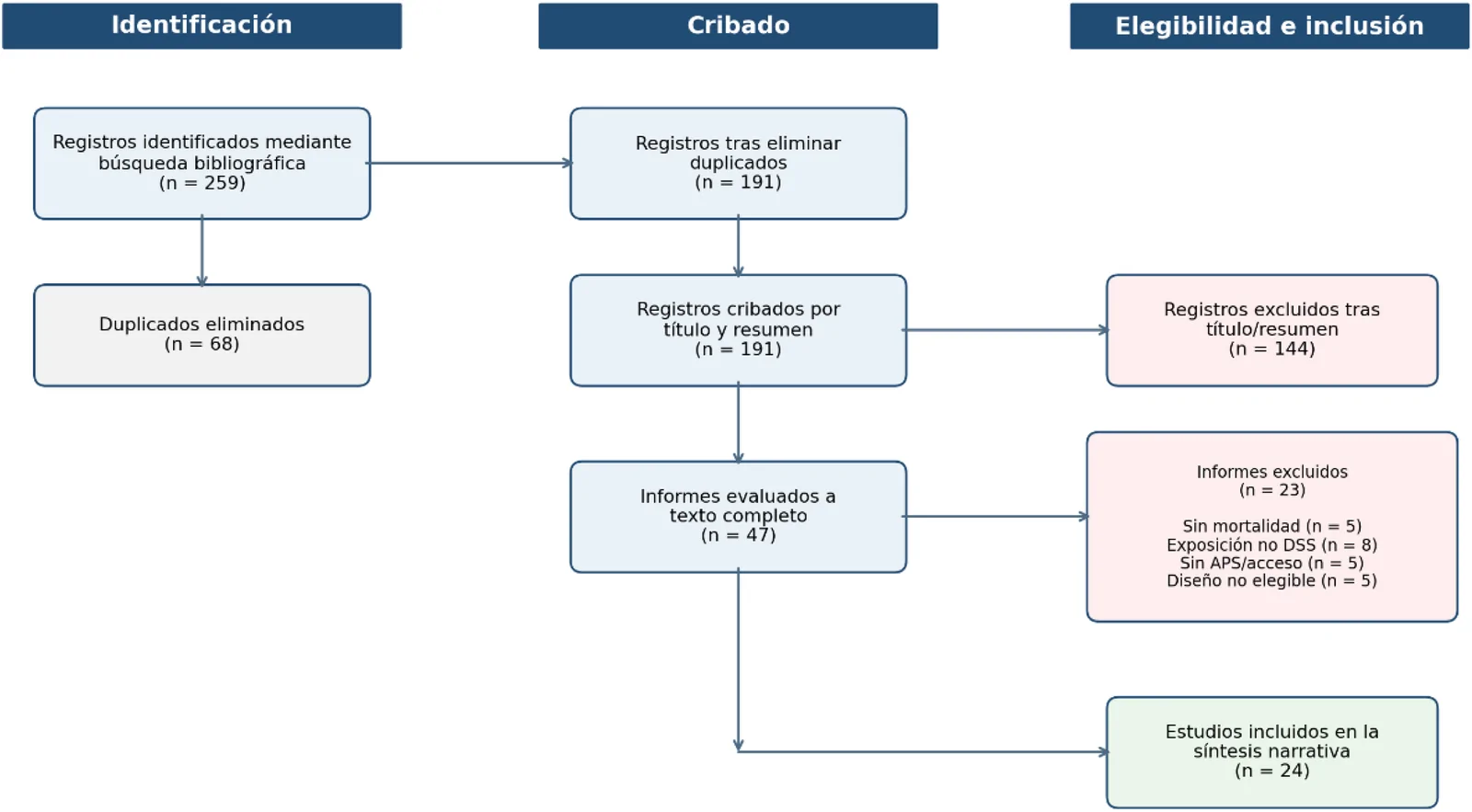
Diagrama de flujo PRISMA 2020 del proceso de identificación, cribado, elegibilidad e inclusión de estudios. APS: atención primaria de salud; DSS: determinantes sociales de la salud.Fuente: Elaboración propia.

### Características de los estudios incluidos

Catorce estudios se realizaron en Estados Unidos; los 10 restantes procedían de España, Colombia, Brasil, Indonesia, Irán, Corea del Sur, Suiza, Países Bajos o análisis multinacionales. Predominaron los diseños ecológicos (n = 13); los demás incluyeron cohortes, estudios retrospectivos, transversales individuales y vigilancia poblacional. Once estudios utilizaron datos individuales y 13 unidades territoriales o macro. La operacionalización de los determinantes fue amplia e incluyó renta, pobreza, protección social, raza/etnicidad, índices de vulnerabilidad, vivienda, ruralidad y accesibilidad. Trece incorporaron indicadores de APS o de acceso a servicios de forma directa o contextual, mediante densidad profesional, zonas de escasez, penetración de centros comunitarios, contacto asistencial, intervenciones comunitarias o acceso geográfico. Las Tabla 1, Tabla 2 resumen las características y la tabla 3 los principales resultados cuantitativos.

**Tabla 1:** Caracterización detallada de los estudios incluidos

Estudio	Escenario y diseño	Exposición/APS	Desenlace
Lane et al.^10^, 2023	EE. UU. (10 estados); cohorte retrospectiva; individuo	Privación social, raza/etnia, contacto APS	Mortalidad total y hospitalaria COVID-19
Smith et al.^22^, 2022	EE. UU. (Wisconsin); cohorte retrospectiva emparejada; individuo	Cuidado retrasado, fragilidad, Medicaid, APS	Mortalidad total
Ali et al.^3^, 2023	EE. UU. (condados); ecológico longitudinal	Vulnerabilidad social y médicos APS	Mortalidad COVID-19
Lin et al.^4^, 2022	EE. UU. (condados); ecológico espacial	Barreras estructurales y APS	Tasa de mortalidad COVID-19
Neelon et al.^5^, 2021	EE. UU.; ecológico longitudinal	SVI y APS	Muertes COVID-19
Gaglioti et al.^6^, 2021	EE. UU.; ecológico transversal	Población negra y escasez APS	Tasa de muertes COVID-19
Valencia Rendon et al.^23^, 2024	Brasil; cohorte prospectiva; individuo	Vulnerabilidad social y acceso	Mortalidad intrahospitalaria
Cifuentes et al.^20^, 2020	Colombia; cohorte retrospectiva; individuo	Condición socioeconómica	Muerte COVID-19
Baltrus et al.^24^, 2021	EE. UU.; ecológico transversal	Población negra y APS	Tasa de muertes COVID-19
Ford et al.^11^, 2022	EE. UU.; ecológico transversal	Cobertura APS	Mortalidad comunitaria
Splinter et al.^25^, 2024	Países Bajos; cohorte longitudinal; individuo	Evitación de cuidados	Mortalidad total
Zadeh et al.^26^, 2024	EE. UU.; ecológico caso-control	Condiciones estructurales	Disparidades de mortalidad
Auderset et al.^27^, 2024	Suiza; vigilancia; individuo	Sexo/género	Muerte COVID-19
Lee et al.^21^, 2024	Corea del Sur; retrospectivo; individuo	Renta y aseguramiento	Mortalidad
Dadgar et al.^28^, 2023	Irán; transversal; individuo	Determinantes sociales	Muerte COVID-19
Liang et al.^12^, 2023	EE. UU.; ecológico	Gasto en salud	Muertes COVID-19
Jones et al.^29^, 2023	EE. UU.; ecológico multinivel	Ruralidad y APS	Tasa de muertes
Leache et al.^19^, 2025	España; cohorte; individuo	Edad y renta	Mortalidad total
Sujarwoto et al.^30^, 2022	Indonesia; transversal; individuo	Acceso geográfico e intervenciones comunitarias	Muerte COVID-19
Ojinnaka et al.^13^, 2021	EE. UU.; ecológico	Densidad de APS y composición racial/étnica	Mortalidad
Islam et al.^7^, 2021	EE. UU.; ecológico	Vulnerabilidad social	Incidencia y mortalidad
Magallón-Botaya et al.^8^, 2021	España; cohorte; individuo	Factores geográficos en APS	Muerte COVID-19
Hashim et al.^9^, 2020	Multinacional; ecológico	Indicadores socioeconómicos	Tasa de mortalidad
Moore et al.^14^, 2020	EE. UU.; ecológico	Infraestructura sanitaria/APS	Mortalidad

APS: atención primaria de salud; EE. UU.: Estados Unidos; SVI: Social Vulnerability Index.

**Tabla 2:** Caracterización sintética de los estudios

Dimensión	Síntesis	Comentario analítico
Distribución geográfica	14 estudios de EE. UU.; 10 de Europa, América Latina, Asia o análisis multinacionales	Base internacional, asimétrica a favor de países de renta alta
Diseño	13 ecológicos; 8 cohortes/retrospectivos; 2 transversales individuales; uno vigilancia retrospectiva	El predominio ecológico exige cautela interpretativa
Unidad de análisis	11 individuales y 13 territoriales/macro	La literatura combina experiencia individual y vulnerabilidad territorial
APS/acceso	13 estudios incorporaron indicadores directos o contextuales de APS/acceso	Los indicadores no fueron estandarizados y abarcaron disponibilidad, cobertura, contacto y accesibilidad

APS: atención primaria de salud; EE. UU.: Estados Unidos.

**Tabla 3:** Síntesis cuantitativa seleccionada de las asociaciones entre determinantes sociales, atención primaria y mortalidad por COVID-19

Estudio/contexto	Eje	Indicador	Medida (IC 95%)	Significación/dirección	Lectura sintética
Leache et al.19, 2025/España	Renta	Renta < 18.000€	aOR 3,29 (2,16-5,03)	Significativa; ↑	Mayor mortalidad; cohorte española
Cifuentes et al.20, 2020/Colombia	Posición social	Estrato muy bajo vs. alto	HR 1,73 (1,48-2,04)	Significativa; ↑	Gradiente socioeconómico
Lee et al.21, 2024/Corea del Sur	Posición social	Medical Aid vs. grupo económico más alto	aOR 1,92 (1,87-1,98)	Significativa; ↑	Mayor mortalidad en menor posición económica
Cifuentes et al.20, 2020/Colombia	Etnicidad	Etnia indígena	HR 1,27 (1,13-1,43)	Significativa; ↑	Asociación contextual específica
Cifuentes et al.20, 2020/Colombia	Etnicidad	Etnia afrocolombiana	HR 1,01 (0,96-1,08)	p = 0,613; —	Sin asociación
Islam et al.7, 2021/EE. UU.	Vulnerabilidad	SVI, quintil mayor vs. menor	RR 2,42 (2,22-2,64)	Significativa; ↑	Fuerte gradiente territorial
Magallón-Botaya et al.8, 2021/España	Territorio	Área suburbana vs. rural	HR 1,31 (1,04-1,66)	Significativa; ↑	Geografía y mortalidad
Sujarwoto et al.30, 2022/Indonesia	Acceso/comunidad	Intervenciones comunitarias de salud	aOR 0,98 (0,97-1,00)	p = 0,008; ↓	Asociación inversa por unidad adicional
Baltrus et al.24, 2021/Georgia, EE. UU.	Déficit APS	Zona con escasez de profesionales	RR 1,74 (1,00-3,00)	p = 0,049; ↑	Mayor mortalidad con déficit profesional
Gaglioti et al.6, 2021/EE. UU.	Déficit APS	PCHPSA	RR 1,004 (0,910-1,106)	p = 0,94; —	Sin asociación a escala nacional
Ojinnaka et al.13, 2021/Texas, EE. UU.	Densidad APS	Tasa de médicos de APS	OR 0,93 (0,89-0,99)	Significativa; ↓	Menor probabilidad de registrar al menos una muerte
Ford et al.11, 2022/4 ciudades, EE. UU.	Cobertura APS	Penetración de FQHC, modelo ajustado	Coeficiente de regresión −185,0	p < 0,01; ↓	Asociación inversa tras ajuste
Liang et al.12, 2023/EE. UU.	Salud pública	Gasto público, tercil mayor	−21 muertes/100.000 (−32 a −10)	Significativa; ↓	Infraestructura de salud pública, no APS específica
Lane et al.10, 2023/EE. UU.	Vía indirecta	Vacunación completa	OR 0,34 (0,27-0,43)	Significativa; ↓	Mortalidad por todas las causas; vía indirecta
Splinter et al.25, 2024/Países Bajos	Acceso	Evitación de cuidados	HR 1,30 (1,01-1,67) → 0,93 (0,71-1,20)	Se atenúa; —	Asociación explicada parcialmente por vulnerabilidad basal

Las medidas se presentan tal como fueron reportadas y no son directamente comparables. No se realizó metaanálisis por la heterogeneidad clínica y metodológica.

aOR: odds ratio ajustada; APS: atención primaria de salud; FQHC: centro de salud federalmente cualificado; HR: hazard ratio; IC 95%: intervalo de confianza del 95%; PCHPSA: zona con déficit de profesionales de atención primaria; RR: razón de tasas o riesgos; SVI: Social Vulnerability Index.

### Resultados cuantitativos según los principales determinantes sociales

Las medidas de asociación fueron heterogéneas y no combinables. Los resultados se presentan tal como fueron reportados, distinguiendo magnitud, dirección y significación estadística (tabla 3).

La mayoría de los estudios sobre renta, posición socioeconómica o pobreza informó mayor mortalidad con mayor privación. En Navarra (España), una cohorte poblacional de 431.091 personas mostró mayor mortalidad entre quienes tenían renta anual < 18.000 € (aOR 3,29; IC 95% 2,16-5,03), mientras que otros estudios sobre privación mostraron una dirección concordante^19^. En Colombia se observó un gradiente frente al estrato alto: HR 1,73 (IC 95% 1,48-2,04) para el estrato muy bajo, 1,61 (1,38-1,87) para el bajo y 1,34 (1,16-1,56) para el medio-bajo; el estrato medio no alcanzó significación (HR 1,16; IC 95% 0,99-1,36; p = 0,059)^20^. En Corea del Sur, los beneficiarios de Medical Aid presentaron mayor riesgo de muerte que el grupo económico más alto (aOR 1,92; IC 95% 1,87-1,98)^21^. En análisis ecológicos estadounidenses, algunos indicadores de pobreza perdieron significación tras el ajuste por otras características territoriales^6^, ^24^, lo que muestra que las asociaciones socioeconómicas dependen de la especificación del modelo y de la escala analítica.

En Estados Unidos, los estudios mostraron asociaciones entre composición racial y mortalidad, aunque varias estimaciones tuvieron magnitudes próximas a la unidad. En Georgia se obtuvo un leve incremento de mortalidad por cada aumento de un punto porcentual en población negra (RR 1,03; IC 95% 1,01-1,05)^24^. Otros análisis nacionales y rurales mostraron una dirección similar^6^, ^26^, pero estas medidas no implican por sí solas una gran magnitud poblacional. En Texas, la composición hispana y afroamericana se relacionó con mayores tasas de mortalidad en los modelos ajustados, mientras que la asociación racial dejó de ser significativa para la probabilidad de registrar al menos una muerte después de incorporar acceso sanitario y otras características del condado^13^. Fuera de Estados Unidos el patrón no fue homogéneo: en Colombia, la etnia indígena se asoció con mayor mortalidad (HR 1,27; IC 95% 1,13-1,43), mientras que la categoría afrocolombiana no mostró asociación (HR 1,01; IC 95% 0,96-1,08; p = 0,613)^20^.

### Vulnerabilidad territorial, vivienda y barreras geográficas

La vulnerabilidad territorial mostró asociaciones de mayor magnitud en varios estudios. En 3.141 condados estadounidenses, el quintil de mayor vulnerabilidad social presentó RR 2,42 (IC 95% 2,22-2,64) frente al menor, con un gradiente dosis-respuesta a través de los quintiles^7^. En Aragón, el área suburbana se asoció con mayor mortalidad que la rural (HR 1,31; IC 95% 1,04-1,66)^8^. En Indonesia, una mayor distancia al hospital de referencia se asoció con mortalidad, mientras que las intervenciones comunitarias de salud mostraron una asociación inversa^30^. En Colombia, el área rural dispersa se asoció con menor mortalidad (HR 0,83; IC 95% 0,76-0,91)^20^. En condados rurales estadounidenses, la desventaja observada se modificó sustancialmente al ajustar por composición demográfica, pobreza, educación, vacunación y salud poblacional^29^. En conjunto, los resultados muestran desigualdades espaciales persistentes, pero no un gradiente rural-urbano uniforme entre contextos.

Los indicadores de APS y acceso mostraron asociaciones heterogéneas y dependientes de la escala. En Georgia, los condados con déficit de profesionales de APS presentaron mayor mortalidad (RR 1,74; IC 95% 1,00-3,00; p = 0,049), mientras que el mismo indicador no se asoció con mortalidad en un análisis nacional estadounidense^6^, ^24^. En Texas, una mayor tasa de médicos de APS se asoció con menor probabilidad de que el condado registrara al menos una muerte por COVID-19 (OR 0,93; IC 95% 0,89-0,99)^13^. En 4 ciudades estadounidenses, el modelo ajustado mostró una asociación inversa entre penetración de centros de salud federalmente cualificados y mortalidad (coeficiente de regresión −185,0; p < 0,01)^11^. En este último estudio, la dirección del coeficiente cambió tras el ajuste, por lo que su interpretación depende de la modelización y no debe expresarse como una reducción causal de muertes. Un mayor gasto público local en salud se asoció con 21 muertes menos por 100.000 habitantes (IC 95% −32 a −10), aunque este indicador representa infraestructura de salud pública y no APS específicamente^12^. En beneficiarios de Medicare, más visitas de APS se asociaron con mayor vacunación; a su vez, la vacunación completa se asoció con menor mortalidad por todas las causas (OR 0,34; IC 95% 0,27-0,43), configurando una vía indirecta^10^. La evitación de cuidados se asoció inicialmente con mortalidad por todas las causas (HR 1,30; IC 95% 1,01-1,67), pero se atenuó tras ajustar por salud autopercibida (HR 0,93; IC 95% 0,71-1,20)^25^.

En conjunto, los estudios que incorporaron APS o acceso muestran asociaciones compatibles con un papel mitigador parcial, pero con resultados dependientes del indicador y de la escala geográfica. Ningún diseño permitió atribuir causalmente una reducción de mortalidad a la APS de forma aislada. Los resultados más consistentes correspondieron a disponibilidad profesional, penetración de centros comunitarios y continuidad/acceso, mientras algunos indicadores fueron nulos o indirectos^6^, ^10^, ^11^, ^12^, ^13^, ^24^, ^25^, ^30^.

### Evaluación de la certeza de la evidencia mediante GRADE

La certeza fue moderada para la asociación entre baja renta/pobreza y mayor mortalidad, por convergencia entre estudios individuales y territoriales en diferentes países^19^, ^20^, ^28^. Fue baja para racialización/segregación, vulnerabilidad territorial y fortaleza de la APS, debido al predominio de diseños ecológicos, heterogeneidad de la medición, indirectness, imprecisión de algunas estimaciones y escasez de medidas directas de APS (tabla 4)^4^, ^6^, ^7^, ^8^, ^11^, ^12^, ^13^, ^14^, ^24^, ^26^.

**Tabla 4:** Síntesis GRADE de la certeza de la evidencia por proposición analítica

Proposición analítica	Base empírica	Hallazgo sintético	Limitaciones principales	Certeza
Baja renta, pobreza y protección social precaria se asocian con mayor mortalidad por COVID-19	Estudios individuales y ecológicos en múltiples países	Asociación consistente con gradiente social	Heterogeneidad de indicadores y confusión residual	Moderada
Racialización y desigualdades étnico-raciales se asocian con mortalidad en contextos específicos	Estudios ecológicos y multivariables, sobre todo de EE. UU.	Dirección frecuente en EE. UU.; magnitudes pequeñas en varios modelos y patrón no homogéneo fuera del país	Indirectness, medición heterogénea y especificidad contextual	Baja
Vulnerabilidad territorial, vivienda precaria y barreras geográficas se asocian con mortalidad	Estudios territoriales y de accesibilidad	Concentración espacial de mortalidad en áreas vulnerables	Falacia ecológica y heterogeneidad de exposiciones	Baja
Mayor disponibilidad y acceso a APS pueden mitigar parcialmente mortalidad o desigualdades	Estudios con indicadores directos y contextuales de APS/acceso	Señales protectoras heterogéneas y dependientes de escala	Pocos estudios con medida directa de APS; ausencia de estandarización	Baja

APS: atención primaria de salud; EE. UU.: Estados Unidos.

## Discusión

### Limitaciones

El predominio de estudios ecológicos exige cautela por falacia ecológica y confusión residual; la heterogeneidad de exposiciones y desenlaces impidió la realización de metaanálisis. La APS fue medida directamente en pocos estudios y, con frecuencia, mediante indicadores de acceso o contexto. La concentración de evidencia en países de renta alta limita la generalización. En particular, 14 de 24 estudios se realizaron en Estados Unidos: los hallazgos sobre composición y segregación racial reflejan configuraciones históricas, institucionales y residenciales específicas y no deben extrapolarse automáticamente. La raza debe interpretarse como marcador social y territorial, no biológico. La ausencia de asociación para la categoría afrocolombiana, frente a asociaciones de pequeña magnitud en estudios estadounidenses, refuerza la necesidad de validación contextual^6^, ^20^, ^24^.

Los hallazgos confirman que la mortalidad por COVID-19 siguió líneas históricas de desigualdad social y territorial. La posición socioeconómica desfavorable se asoció con mayor mortalidad en distintos países y sistemas sanitarios^19^, ^20^, ^21^, lo que evidencia que la cobertura formal no elimina diferencias materiales de exposición, vivienda, empleo y capacidad de protección. La convergencia de resultados individuales y territoriales sustenta este eje como el de mayor certeza relativa de la revisión.

En Estados Unidos, la composición racial se asoció de forma consistente con la mortalidad, pero varias estimaciones ajustadas fueron pequeñas. Por ello, la significación estadística no debe equipararse automáticamente con relevancia clínica o poblacional. Estas asociaciones adquieren sentido cuando se interpretan junto con pobreza, segregación residencial, acceso a servicios y racismo estructural, y no como efectos biológicos de la raza^4^, ^6^, ^13^, ^24^, ^26^. La ausencia de una asociación equivalente para la categoría afrocolombiana ilustra que las categorías raciales y sus magnitudes no son transferibles entre sociedades^20^.

La vulnerabilidad territorial mostró asociaciones de mayor magnitud en algunos análisis, como el gradiente del Social Vulnerability Index^7^, reforzando que el territorio concentra exposiciones, recursos y capacidad institucional. Los resultados rurales, sin embargo, variaron según país y ajuste, lo que desaconseja considerar la ruralidad como riesgo intrínseco y favorece analizar los mecanismos concretos de accesibilidad, composición social y oferta de servicios^8^, ^29^, ^30^.

La APS parece modificar parcialmente la relación entre determinantes sociales y mortalidad, pero la evidencia no demuestra un efecto causal aislado. Los hallazgos más directamente vinculados con la APS proceden de indicadores de disponibilidad profesional y penetración de centros: el déficit de profesionales en Georgia se asoció con una magnitud relevante de mortalidad, mientras que la mayor densidad de médicos en Texas mostró una asociación inversa y la penetración de FQHC presentó una señal protectora después del ajuste^11^, ^13^, ^24^. En contraste, el indicador nacional de escasez de APS no mostró asociación^6^, evidenciando heterogeneidad por escala y contexto.

Los estudios de vacunación, evitación de cuidados y gasto público amplían el marco, pero representan vías indirectas o infraestructura sanitaria más amplia y deben interpretarse como evidencia contextual, no como estimaciones directas del efecto de la APS^10^, ^12^, ^25^. Para sistemas universales y para Iberoamérica, los resultados apoyan una APS territorializada, comunitaria y sensible a desigualdades, articulada con políticas sociales, vigilancia y salud pública.

### Directrices para futuras investigaciones

Se requieren estudios con medidas directas y comparables de APS, análisis de mediación y mayor representación de países de renta baja y media. La preparación para futuras emergencias debe combinar APS territorializada, vigilancia sensible a desigualdades, infraestructura de salud pública y políticas redistributivas.

## Conclusiones

La mortalidad por COVID-19 estuvo socialmente estratificada y territorialmente modulada. Pobreza, vulnerabilidad territorial, condiciones de vivienda y procesos de racialización fueron ejes recurrentes. La APS mostró asociaciones compatibles con atenuación parcial cuando existían mayor disponibilidad profesional, acceso y continuidad, pero no eliminó el gradiente social.

La resiliencia frente a futuras emergencias requiere integrar APS territorializada, sistemas universales consistentes y políticas redistributivas capaces de actuar sobre las condiciones que producen vulnerabilidad^1^, ^2^.

**Lo conocido sobre el tema**

– La literatura ha mostrado asociaciones entre desigualdad social y peores desenlaces por COVID-19 en múltiples contextos.
– El papel específico de la atención primaria como factor mitigador de la mortalidad pandémica permanece insuficientemente sintetizado.
– La heterogeneidad de indicadores y diseños dificulta una apreciación integrada de la relación entre determinantes sociales, atención primaria y mortalidad.

**Qué aporta este estudio**

– La atención primaria muestra asociaciones compatibles con una mitigación parcial del gradiente social de mortalidad, con heterogeneidad según indicador y escala.
– La mortalidad pandémica se concentró en territorios donde pobreza, vulnerabilidad, barreras geográficas y fragilidad institucional se superpusieron.
– La preparación frente a futuras emergencias requiere fortalecer una atención primaria territorializada e integrar políticas redistributivas.

## Financiación

Coordenação de Aperfeiçoamento de Pessoal de Nível Superior (CAPES). La fuente de financiación no tuvo participación en el diseño del estudio, en la recopilación, análisis e interpretación de datos, en la redacción del informe ni en la decisión de enviar el artículo para su publicación.

## Consideraciones éticas

No se precisó aprobación por un comité de ética por tratarse de una revisión sistemática de literatura publicada. Revisión registrada en PROSPERO, con número CRD42024502678.

## Declaración sobre el uso de la IA generativa y de las tecnologías asistidas por la IA en el proceso de redacción

Durante la preparación de este trabajo los autores utilizaron Claude (Anthropic) y ChatGPT (OpenAI) para apoyar la organización, la revisión lingüística y la presentación de la respuesta editorial. Tras utilizar estas herramientas, los autores verificaron las fuentes, revisaron y editaron el contenido y asumen plena responsabilidad por la exactitud, la originalidad y el contenido final de la publicación.

## Conflicto de intereses

Los autores declaran ausencia de conflicto de intereses.
